# A Beautiful Bind: Phage Display and the Search for Cell-Selective Peptides

**DOI:** 10.3390/v17070975

**Published:** 2025-07-12

**Authors:** Babak Bakhshinejad, Saeedeh Ghiasvand

**Affiliations:** 1 Cluster for Molecular Imaging, Department of Biomedical Sciences, University of Copenhagen, 2200 Copenhagen, Denmark; 2 Department of Clinical Physiology and Nuclear Medicine, Copenhagen University Hospital-Rigshospitalet, 2100 Copenhagen, Denmark; 3 Department of Biology, Faculty of Sciences, Malayer University, Malayer 6571995863, Iran; 4 Department of Biotechnology, Faculty of Interdisciplinary Sciences and Technologies, Malayer University, Malayer 6571995863, Iran

**Keywords:** affinity maturation, biopanning, cell-selective ligands, combinatorial libraries, cyclic peptides, mutagenesis, next-generation sequencing, phage display, secondary libraries, sequence space

## Abstract

Phage display has advanced the discovery of peptides that selectively bind to a wide variety of cell surface molecules, offering new modalities to modulate disease-related protein–protein interactions (PPIs). These cell-binding peptides occupy a unique pharmaceutical space between small molecules and large biologics, and their growing popularity has opened up new avenues for targeting cell surface proteins that were previously considered undruggable. This work provides an overview of methods for identifying cell-selective peptides using phage display combinatorial libraries, covering in vitro, ex vivo, and in vivo biopanning approaches. It addresses key considerations in library design, including the peptide conformation (linear vs. cyclic) and length, and highlights examples of clinically approved peptides developed through phage display. It also discusses the on-phage chemical cyclization of peptides to overcome the limitations of genetically encoded disulfide bridges and emphasizes advances in combining next-generation sequencing (NGS) with phage display to improve peptide selection and analysis workflows. Furthermore, due to the often suboptimal binding affinity of peptides identified in phage display selections, this article discusses affinity maturation techniques, including random mutagenesis and rational design through structure–activity relationship (SAR) studies to optimize initial peptide candidates. By integrating these developments, this review outlines practical strategies and future directions for harnessing phage display in targeting challenging cell surface proteins.

## 1. Introduction

Forty years ago, in the spring of 1985, a paper was published in *Science* that promised the birth of a new technology [1]. This novel technical achievement, which was later called phage display, laid the foundation for numerous scientific discoveries in the years that followed. Phage display involves expressing foreign peptides or proteins on the surface of bacteriophages. In its four-decade-long existence, this molecular display technique has made a significant contribution to the field of peptide discovery, providing high-diversity, genetically encoded libraries [2,3] that can be searched to discover binders to a variety of biologically relevant targets. Cell surface proteins are one of the most important biological targets widely used in phage display studies. In this context, phage display enables the identification of peptide ligands capable of binding to proteins involved in critical biological processes and disease pathways. The selective enrichment of high-affinity peptides is achieved through the affinity selection of phage display libraries on cell surface proteins. Cell surface protein-binding peptides, as short amino acid sequences selected from phage display combinatorial libraries, allow scientists to probe, map, and ultimately manipulate signal transduction pathways. By performing the library selection on cell surface proteins and receptors that are either recombinantly produced or are present on intact cells and tissues, phage display offers a highly adaptable platform for isolating peptides that selectively bind to biomolecules associated with a broad range of diseases. Applications of this technology span different scientific areas, such as oncology, immunology, metabolomics, and neurology. In addition to direct biological functions on cells, cell-binding peptides identified by phage display also hold great potential for developing targeted drug delivery [4,5] and imaging [6,7] modalities. Today, phage display has matured into a powerful approach for developing peptides with potential diagnostic and therapeutic applications [8].

The capacity of phage display to identify selective binders to diverse cell surface proteins has not only advanced our understanding of cell biology and disease mechanisms but has also had a profound impact on the identification of therapeutic peptides [9,10,11]. This interface between peptide selection and clinical translation introduces phage display as a powerful discovery engine that drives the development of next-generation peptide-based therapeutics, constituting a dynamic and rapidly evolving area of biomedical research. Therapeutic peptides represent a distinct class of pharmacological agents with molecular weights typically ranging from 500 to 5000 Daltons and occupy a unique pharmaceutical space between small molecules and large biologics [12]. The research on therapeutic peptides began with early studies on natural human hormones—such as insulin, oxytocin, vasopressin, somatostatin, and the gonadotropin-releasing hormone—all of which are short peptides, and the investigation of their specific physiological functions in the body [13]. Insulin was the first commercialized peptide drug, developed by Eli Lilly and Company, and received FDA approval in 1923. The groundbreaking success of insulin as a drug sparked widespread public interest in peptide therapeutics, paving the way for other animal-derived peptide drugs to enter clinical applications. Notable examples include the adrenocorticotropic hormone and calcitonin, which became important early contributors to the peptide pharmacopeia. The second half of the 20th century (from the 1950s to the 1990s) witnessed the discovery and characterization of an increasing number of peptide hormones and their receptors with therapeutic potential. The onset of the 21st century marked a new era in peptide drug development, driven by substantial breakthroughs in structural biology, omics technologies, recombinant methodologies, and novel synthetic and analytical methods. Since 2000, over 30 non-insulin peptide drugs have received regulatory approval globally [14]. In recent decades, peptides have gained increasing recognition as biologically active and therapeutically valuable molecules. Their favorable pharmacological characteristics and intrinsic features have positioned them as attractive candidates in modern drug development pipelines. Peptides present multiple advantages for clinical applications, such as high specificity, favorable cell and tissue penetration, easy synthesis, low production costs, reduced immunogenicity, and amenability to modifications [12,15,16,17]. Over time, the therapeutic applications of peptides have progressed steadily and continue to evolve with ongoing advancements in drug design and treatment strategies. The development of peptide drugs remains a hot topic in pharmaceutical research. The soaring popularity of peptide therapeutics has opened up new avenues for creating novel platforms that can modulate undruggable targets associated with a wide range of diseases [18,19]. The current review aims to provide an overview of the current knowledge on phage display and its application for the discovery and refinement of peptides binding to cell surface proteins. We begin by discussing the foundational principles of phage display technology and the molecular architecture of peptide libraries, with a particular focus on the different conformations (linear vs. cyclic) and lengths of displayed peptides during library design. We then explore the various modes of target presentation in the affinity selection of phage display peptide libraries and investigate how the biological context of the target impacts the selection outcome. Finally, we discuss in detail the various approaches that are employed to mature the binding affinity of peptides isolated from phage display libraries, making them optimal for the potential translation into the clinic.

## 2. Phage Display and Combinatorial Peptide Libraries

**Figure 1:**
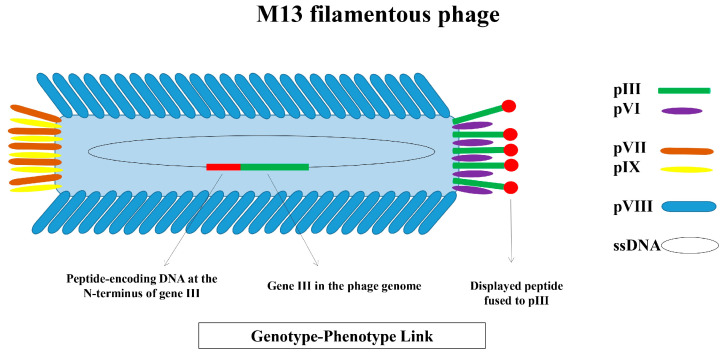
The structure of the M13 filamentous phage, and the schematic illustration of the genotype–phenotype link M13 is a rod-shaped bacteriophage with a single-stranded DNA (ssDNA) genome encapsulated by five coat proteins. Four minor coat proteins (pIII and pVI at one end; pVII and pIX at the other) are present in 3 to 5 copies per virion, while the major coat protein (pVIII) is present in approximately 2700 copies and covers the phage body. M13 is the most widely used bacteriophage for the surface display of ligands, including peptides. In phage display, foreign peptide-encoding DNA is typically inserted at the N-terminus of gene III, resulting in the expression of the peptide on the phage surface while fused to the pIII coat protein. This physical linkage between the encoding DNA (genotype) and the displayed peptide (phenotype) enables the sequence identification of peptides displayed on phages isolated during biopanning.

Bacteriophages, also known as phages, are viruses that specifically infect bacteria. Phage display was born in the mid-1980s, when George Smith introduced the concept that filamentous bacteriophage can serve as an expression vector capable of displaying antigens cloned into the phage genome on the virion surface [1]. He inserted foreign DNA fragments into gene III of the f1 phage and demonstrated that, as the phage assembles inside the host bacterium and is extruded from the cell, the foreign sequences are displayed on the phage surface as fusions to the pIII coat protein without disrupting the infectivity of the phage particle. The peptides encoded by the inserted sequences were in an accessible form and could directly interact with a target of interest. Since then, various phages, such as T4 [20], T7 [21], λ (lambda) [22], MS2 [23], and Qβ [24], have been used for display purposes. However, filamentous phages of the Ff family, including M13, fd, and f1, remain the most widely adopted platforms for the display of biomolecules, with M13 as the most predominant in the field [25,26]. The dominance of Ff bacteriophages over other phages for ligand display is attributed to their ability to achieve titers higher than those of other known phages, an efficient transformation that enables the generation of diverse libraries, and the robust stability of their virions under high temperatures, detergents, and extreme pH conditions. These properties collectively facilitate a broad range of biopanning conditions for the affinity selection of phage display libraries [26]. Additionally, these phages exhibit great genetic flexibility, making them well-suited for genetic engineering. This flexibility allows the enlargement of the genome size to be accompanied by a proportional increase in both the length of the phage particle and the number of pVIII molecules. As a result, the encapsulated DNA does not impose a limitation on the size of the phage particle [27]. These filamentous phages infect F plasmid-containing Gram-negative bacteria, like *Escherichia coli*, and follow a non-lytic life cycle. After the replication of the phage DNA, the translation of phage proteins, and the assembly of phage particles, new virions are released from infected bacteria without causing the lysis of the host cell. Although all five coat proteins of the M13 phage have been used for the surface presentation of foreign amino acids, pIII (one of the minor coat proteins with 406 residues) and pVIII (the major coat protein with 50 residues) are the most important coat proteins that have been broadly employed for peptide display [28]. The integration of the idea of combinatorial chemistry [29] with phage display led to the generation of phage display peptide libraries [2,3]. These combinatorial phage libraries house a large repertoire of peptide sequences that can be searched to identify ligands with desired binding characteristics. In phage display libraries, the physical linkage between the displayed peptide and the nucleotide sequence that encodes it, known as the genotype–phenotype link, is a foundational pillar of the technique that enables the sequence identification of displayed peptides with sought-after binding features [26,30]. Figure 1 illustrates the general structure of the M13 phage with its coat proteins and the physical linkage between the genotype and phenotype in phage display.

Peptides can adopt different structural conformations on the virion surface, which must be carefully considered in the design of phage display libraries. Linear libraries consist of peptides of varying lengths, typically ranging from fewer than 10 to over 40 amino acids. The linear form offers structural simplicity, allowing an easier direct interaction with target molecules. In contrast, cyclic libraries are generated by introducing cysteine residues into the peptide sequences, resulting in the formation of intramolecular disulfide bonds [31]. These covalent linkages create looped structures and diverse folded conformations in the displayed peptides. Peptides with such secondary structures provide advantages for target binding. The conformational constraint imparted by disulfide bridges often enhances the binding affinity and selectivity of displayed peptides [32,33], which is particularly beneficial when targeting complex binding sites or increasing the peptide half-life. Disulfide bridges are a key structural feature in many natural miniproteins, such as scorpion venoms and plant toxins [34]. To date, dozens of cyclic peptides have received FDA approval. A comprehensive list of FDA-approved cyclic peptide drugs in the past two decades and their respective indications can be found in [35]. More specifically, several clinically approved peptide drugs with cyclic structures have been developed employing phage display technology. Notably, pegcetacoplan, used for the treatment of paroxysmal nocturnal hemoglobinuria (PNH), is a pegylated cyclic peptide targeting and inhibiting complement protein C3, which was discovered and optimized through phage display [36]. Ecallantide, approved for the treatment of acute hereditary angioedema (HAE), is a human plasma kallikrein inhibitor containing three intramolecular disulfide bonds identified through selection of phage display peptide libraries [37]. In addition to the cyclic peptide drugs already approved, several promising candidates are in late-stage clinical development and may receive regulatory approval in the near future. These examples illustrate the expanding role of cyclic peptides in modern drug discovery, which offer robust and effective therapeutic options across a variety of medical areas. The advent and further advancements in display technologies, such as phage display, along with the development of sophisticated synthetic chemistry approaches (e.g., automated synthesis platforms) have played a significant role in discovering and optimizing cyclic peptides. While disulfide bridges offer clear structural and functional advantages by constraining peptide conformation and enhancing binding affinity and specificity, their incorporation into phage-displayed peptide libraries is not without challenges. A key limitation is the susceptibility of disulfide bonds to reducing intracellular environments [38,39], which can lead to bond cleavage and conformational instability. The reducing conditions in the cytoplasm of host bacteria can disrupt the structural integrity of cyclic peptides. Additionally, the sensitivity of disulfide bridges to reducing environments poses a challenge for evaluating hits from such libraries in cell-based assays [39]. Cyclic conformation can also impair the propagation efficiency of phage particles [40], potentially due to rigid or bulky structures that are less compatible with the phage coat proteins. This incompatibility may compromise the proper phage assembly during the amplification of phage display libraries. Collectively, these effects can reduce the library diversity and ultimately impact the outcome of the selection in phage display experiments. Thus, while cyclic peptides offer structural and functional benefits, a careful design is required to balance the stability and library diversity in phage display. Peptides displayed on phages can also be cyclized by applying various chemical strategies. The development of methods for chemically modifying peptides displayed on phages has significantly expanded the range of accessible cyclic peptide architectures, thereby enhancing the structural diversity and pharmacological potential of phage display libraries [41]. Chemical cyclization on phages was first applied to generate bicyclic peptides [42]. These molecules are composed of two macrocyclic rings, formed by chemically linking specific residues within the peptide sequence. Compared to monocyclic peptides of a similar molecular weight, bicyclic peptides exhibit greater conformational rigidity, which often results in an enhanced binding affinity for their targets and an increased resistance to proteolytic degradation [43]. Phage-displayed peptides can be chemically converted into bicyclic structures by reacting three cysteine residues in the peptide with a reagent containing three thiol-reactive groups. Reagents were selected to ensure efficient cyclization through cysteine residues while preserving the structural integrity of phage coat proteins, thereby maintaining the phage infectivity. This strategy was used to induce bicyclization by treating a phage library, displaying the peptide with the ACX_6_CX_6_CG format fused to the p3 coat protein, with 1,3,5-tris(bromomethyl)benzene (TBMB). The subsequent biopanning of this library against the proteases plasma kallikrein and cathepsin G led to the isolation of bicyclic peptides with nanomolar-range inhibitory constants [42]. Beyond TBMB, other chemical reagents have been employed to generate cyclic peptides on phages. For instance, azobenzene linkers have been used to generate and identify light-responsive cyclic peptide ligands [44,45], while ortho-phthalaldehyde (OPA) has enabled the formation of isoindole-bridged cyclic peptides, which have been successfully applied to isolate ligands targeting several therapeutically relevant proteins [46].

The length of displayed peptides is another critical factor in the design of phage display libraries [47]. Even short peptides, when fully randomized, yield a library size that far exceeds the capacity of phage display systems. The theoretical sequence space increases exponentially with the peptide length. Given the 20 naturally occurring amino acids, the total number of possible sequences for a peptide of length N is 20^N^. However, the real sequence space that can be covered by a phage display library is typically around 10^8^–10^9^ unique variants, due to limitations in the transformation efficiency during the library construction [48]. This restriction means that the complete coverage of the sequence space is only achievable for peptides with up to six fully randomized positions (hexamer libraries). For peptides of seven or more residues, only a small fraction of the total sequence space is represented by the library. The fraction of the theoretical sequence space that can be represented by the library is dramatically reduced with the increased length of displayed peptides (Table 1). For example, a 20-mer library encompasses such a vast theoretical sequence space that only a minuscule portion (9.54 × 10^−18^) of the sequence space can be covered in a peptide library displayed on the phage.

Both the length and conformation of displayed peptides have also been demonstrated to significantly affect the propagation capacity of phage clones, potentially leading to unbalanced changes in the composition and diversity of the library and consequently a biased selection of target-binding peptides during biopanning [40]. Therefore, the careful balancing of the peptide length and conformational complexity is crucial to maximize the functional diversity represented in the library and to minimize the selection bias during biopanning.

## 3. Affinity Selection of Phage Display Peptide Libraries Through Biopanning

Biopanning is the core selection process used to isolate target-specific peptides from phage display libraries. It relies on affinity-based interactions between the target molecule and a vast phage-displayed peptide repertoire, often comprising millions of unique variants [3,49]. The process begins with the incubation of the phage library with the target of interest. Phage particles that do not bind, or bind weakly, are removed through a series of stringent washing steps. In contrast, strongly bound phages are retained and subsequently eluted. The eluted phage pool is then amplified by infecting the host bacteria, enabling the replication and secretion of new virions into the culture medium. This process is typically repeated over multiple rounds, enriching the population of phages displaying peptides with a high affinity and specificity for the target. After the enrichment, the DNA is extracted from the phage pool and sequenced to identify the peptide-coding regions. Conventionally, the identification of isolated peptide sequences in the biopanning output has been performed by plating the recovered phage pool and sequencing a limited number of viral plaques that appear on the plate. However, in recent years, a growing number of phage display studies are employing high-throughput sequencing (HTS), also known as next-generation sequencing (NGS), which enables the parallel sequencing of the DNA extracted from thousands to millions of isolated phage clones [50,51,52,53,54,55,56]. NGS-based phage display brings significant advantages over the conventional low-throughput sequencing of the biopanning output, yielding quantitative insights into the peptide composition of phage pools [57,58]. The incorporation of NGS into biopanning has challenged some of the core assumptions underlying phage display selection. For example, analyzing the sequence content of the naïve library by NGS has found a significant bias in the nucleotide and amino acid composition, highlighting that the unselected library is not truly random. Consequently, the peptide enrichment during biopanning is not solely driven by the binding affinity for the target. These deviations from randomness can skew the selection outcomes, leading to the erroneous isolation of false-positive hits or the failure to identify genuine binders [48]. The NGS-based sequencing of millions of phage clones from the biopanning output has also shown that a single round of biopanning can be sufficient to identify target-specific binders, thereby accelerating ligand discovery. Omitting further rounds significantly reduces amplification-associated biases, helping to preserve potentially high-affinity binders that might otherwise be lost [59]. Additionally, NGS enables the identification of rare target-binding peptide motifs and allows for a more accurate determination of consensus sequences and sub-groups of consensus sequences within the selected peptide pool [60]. Another important application of NGS is to enable us to uncover the corruption of the selection output, a phenomenon not captured by conventional Sanger sequencing [58]. Corruption can occur when certain clones become overrepresented during biopanning, not because of the true binding affinity for the target, but due to propagation advantages. These clones dominate the phage pool during biopanning rounds, resulting in the undesirable enrichment and isolation of nonspecific binders. The NGS-assisted detection of such corruption in earlier rounds of selection can inform the decision to terminate the biopanning or halt the downstream validation of isolated peptides. By overcoming some of the major bottlenecks of Sanger sequencing and providing deeper insights into the peptide composition of the selection output, NGS helps avoid the misidentification of nonspecifically enriched target-unrelated peptides (TUPs) and improves the reliability of biopanning findings [58]. The comprehensive profiling of peptide populations by NGS generates large sequence datasets that are ideal for training machine learning (ML) models. These models hold great potential for the computational deconvolution of selection noise (nonspecific binders) vs. true target-specific peptides, as well as the identification of sequence motifs or clusters associated with target binding. Before the biopanning on the target, a target-independent capture and elimination can also be incorporated into the selection protocol, intended to remove binders that interact with off-target molecules present in the selection system. The main objective of this modification, which is called negative or subtractive selection, is to deplete the library of binders to the components of the selection apparatus, thereby minimizing background noise caused by cross-reactivity [61,62,63]. This can be achieved by pre-incubating the library with the solid support containing all the elements of the selection apparatus except the target. Afterward, the subtracted phage supernatant retrieved from the negative selection is collected and used as the input for the positive selection, where the target is exposed to the subtracted library. To introduce a higher level of specificity to the ligand selection, a structurally similar control protein molecule that lacks the desired epitope(s) can be employed during the negative selection to discard library members binding nonspecifically to common features between the target and control molecules. When whole cells expressing the target receptor on their surface are used in library selection, control cells either not expressing or expressing very low levels of the target molecule are included in the negative selection [64]. Despite its benefits, the negative selection can bring down the diversity of the library, potentially eliminating useful candidates if performed in a highly stringent manner.

**Figure 2:**
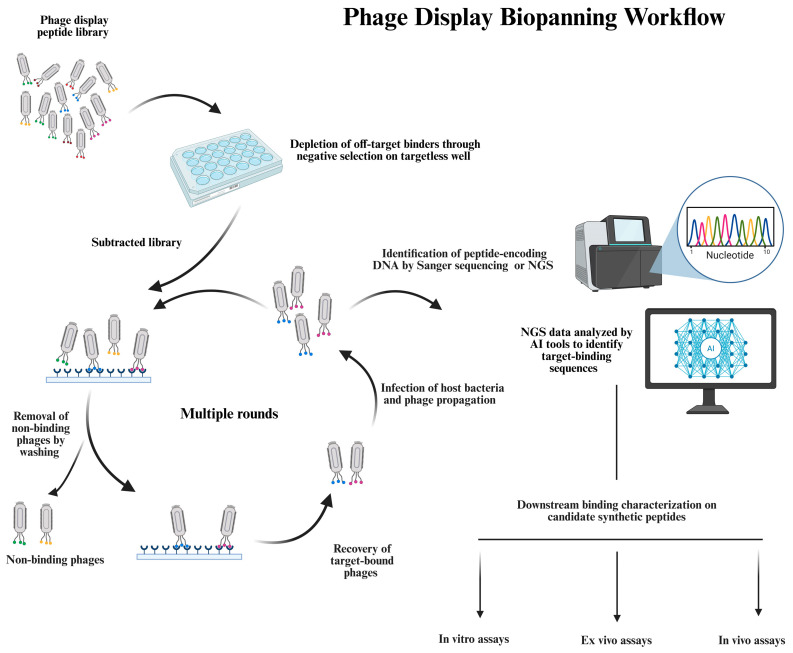
A schematic overview of the phage display biopanning workflow and downstream validation. This figure illustrates the stepwise process of identifying target-specific peptides from a combinatorial phage display library. Initially, the library undergoes negative selection against a targetless solid support to remove phages that bind nonspecifically to the components of the selection system. The resulting subtracted library, depleted of off-target binders and obtained from the negative selection, is employed as input for the positive selection on the target of interest. After incubation, unbound or loosely bound phages are removed by stringent washing steps, and target-bound phages are eluted and then amplified in host bacterial cells to generate an enriched phage pool for subsequent rounds of selection. After multiple rounds of enrichment, the phage pool is subjected to DNA sequencing using either the Sanger approach or NGS. NGS offers a quantitative analysis of the peptide representation within the pool and enables data-driven interpretations using computational tools, such as artificial intelligence (AI) algorithms, to distinguish true binders from selection noise. One or more candidate peptides enriched in biopanning and identified through sequencing are chemically synthesized, and their target binding is investigated out of the phage scaffold through a stepwise workflow, starting with in vitro assays, proceeding to ex vivo analyses, and further advancing to in vivo testing in relevant animal models. This sequential validation ensures functional relevance and minimizes failures in subsequent validation phases, ultimately facilitating the discovery of target-selective peptide ligands.

Once the amino acid sequences of identified peptides are known, the peptides are chemically synthesized, and their target binding properties are initially evaluated using a variety of in vitro assays. Peptides are selected against the target in biopanning while they are displayed on the phage. However, we should make sure that the free (synthetic) peptide retains the same binding capacity toward the target. Therefore, it is necessary to confirm the binding for the target while peptides are chemically synthesized and removed from the phage scaffold. After the initial characterization, peptides that show promising results proceed to ex vivo and in vivo assays. Ex vivo assays assess the peptide binding for the target in biological samples using cells or tissue extracts from animals or humans, providing a more physiologically relevant environment for the binding characterization. The final confirmation of the target-binding capacity before entering clinical trials is conducted in vivo by evaluating the peptide behavior and its target binding in living organisms using animal models. Different assays are employed at each stage—in vitro, ex vivo, and in vivo—of the binding characterization. This stepwise workflow for downstream characterization ensures that only the most promising candidates identified through biopanning advance to further characterization, thereby reducing costs and minimizing unnecessary testing. Figure 2 depicts the phage display biopanning workflow, including the iterative rounds of the library selection on the target, the sequence analysis of the biopanning output to identify candidate peptides with a potential target-binding capacity, and ultimately the downstream characterization to validate the target-binding specificity and affinity of candidate peptides through a variety of in vitro, ex vivo, and in vivo assays.

## 4. Target Presentation in Biopanning

**Figure 3:**
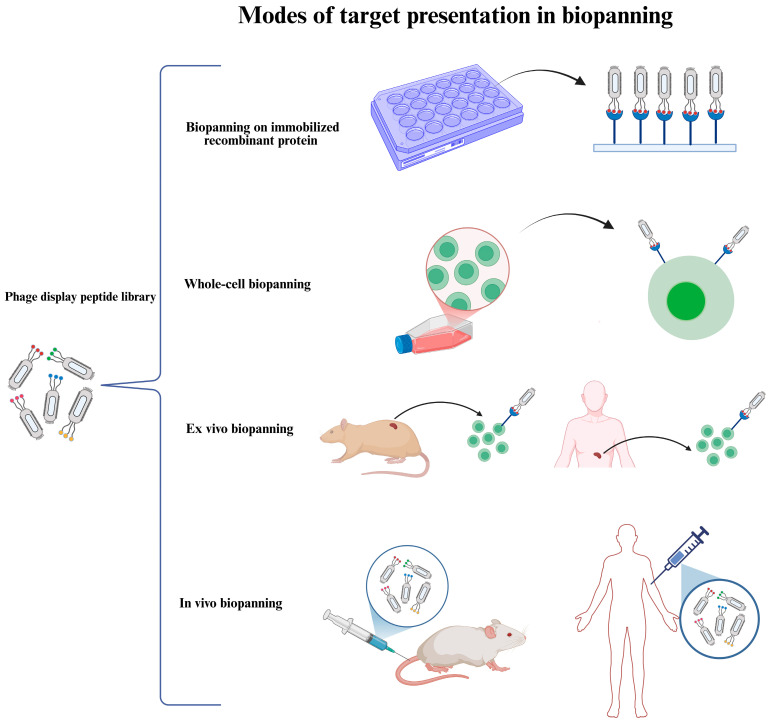
Different modes of target presentation in biopanning The target employed in the selection of phage display peptide libraries might be presented in various modes: (1) directly adsorbed or indirectly immobilized purified recombinant protein (e.g., the extracellular domain of a cell surface protein), (2) present on cultured cells (whole-cell biopanning), (3) present on cells derived from animal or human tissues (ex vivo biopanning), and (4) present within the animal or human body (in vivo biopanning).

The choice of the target type is one of the most critical considerations in designing an effective biopanning strategy, as it directly influences peptide selection outcomes and the physiological relevance. The target employed in biopanning can be presented to the library in different modes (Figure 3). In this section, these modes are discussed critically, highlighting the advantages and disadvantages of each presentation format.

### 4.1. Biopanning on Recombinant Proteins

Due to its convenience, the use of the recombinantly produced cell surface protein is the most widely used approach for the target presentation in biopanning. However, the method utilized to immobilize the recombinant protein has a substantial impact on the success of the selection process [65,66]. The target cell surface protein can be tethered to a variety of solid supports, including multi-well polystyrene plates, polystyrene tubes, Petri dishes, magnetic beads, nitrocellulose surfaces, and monolithic columns [49].

A common strategy involves the direct or passive adsorption of the recombinant protein onto a solid support [66]. Although this method does not require any modification of the protein, it can induce some conformational changes or partial denaturation, resulting in occluded or inaccessible binding sites for ligands [67]. Consequently, some binders selected using this approach may fail to recognize the target cell surface protein in its native, soluble form. An alternative to direct adsorption is solution-phase biopanning, in which the target is first incubated with the phage library in a homogeneous solution, and the resulting phage–target complexes are subsequently captured on plates or magnetic beads. This two-step mode of capture allows for better control over binding kinetics without the complications associated with surface reactions [49]. The affinity capture in this method typically requires the target to carry an affinity tag. One common approach involves biotinylating the target and capturing the phage–target complex on a streptavidin-coated polystyrene plate. Biotin is a widely used affinity tag that captures target molecules based on a high-affinity interaction between biotinylated protein and streptavidin molecules coated on the solid support [3]. The phage–target complex can also be captured using magnetic beads specific for the target. The specificity of beads for the target is created by various affinity tags. For example, if the target protein is fused to affinity tags such as glutathione S-transferase (GST), the maltose-binding protein (MBP), or a polyhistidine tag, the phage–target complex can be captured using glutathione-, amylose-, or nickel-coated beads, respectively. To minimize the nonspecific selection of bead-binding peptides, a negative selection step can be included in the biopanning protocol by pre-incubating the phage library with the bead matrix in the absence of the target protein. Compared to the direct coating method, solution biopanning provides several advantages, including the enhanced accessibility of binding sites on the target, directional presentation of the target to the phage-displayed peptide with minimal disruption to its native conformation, reduced risk of the partial denaturation of the target due to the adsorption to the plastic surfaces, and significantly lower quantities of the target protein required per experiment [30].

### 4.2. Biopanning on Whole Cells

Whole cells can also be employed in the phage display selection. This type of biopanning can be performed on both suspension and adherent cells. In whole-cell biopanning, the target cell surface protein is present on live cells and thus retains its natural conformation, including proper folding, a quaternary structure (for multi-domain proteins), correct post-translational modifications, and interactions with neighboring molecules [68]. In addition, this method eliminates the need for the purification of the target protein. This is particularly advantageous when dealing with transmembrane cell surface receptors, as the purification of these proteins is difficult due to a significant number of hydrophobic residues [69,70]. Whole-cell biopanning is a potent strategy for isolating peptides that bind to cell surface proteins. However, the protocol of whole-cell biopanning can also be adapted to identify internalizing peptides [63,71,72,73]. This can be achieved by increasing the temperature of the library incubation with cells to 37 °C (instead of 4 °C for cell surface-bound phages) to allow for the receptor-mediated endocytosis of phages and their corresponding displayed peptides. It also needs a differential phage recovery that is performed through the surface stripping of target cells to remove extracellularly bound phages and then lysing the cells to release internalized ones. Cell-internalizing peptides facilitate the intracellular transport of molecules and therefore hold great potential for the targeted delivery of therapeutic cargoes into cells. As noted, whole-cell biopanning enables peptide selection in a more relevant cellular context with a preserved cell surface topography. Nevertheless, it deserves to be mentioned that cultured cells used in library selection have undergone a long-term culture along with repeated passaging, which can result in a wide variety of genetic mutations and chromosomal abnormalities. These cell proliferation-associated genotypic changes lead cultured cells to deviate from the original phenotype (a phenomenon known as phenotypic drift) [74,75], which might change the conformation, expression level, and other features of the target protein on the cell surface. Thus, cell-selective peptides identified through biopanning on cultured cells might demonstrate a low reproducibility and poor translational potential.

### 4.3. Ex Vivo Biopanning

Ex vivo target presentation represents another valuable approach in phage display selection. Ex vivo biopanning performed on animal- or patient-derived tissues offers several advantages over whole-cell biopanning [76,77,78]. This approach of library selection avoids the drawbacks associated with the long-term culture and continuous proliferations, providing a more physiologically relevant context due to the presence of a natural tissue architecture, including the extracellular matrix (ECM), cell–cell interactions, and vascular structures that are lost in whole-cell biopanning. These features increase the translational relevance of peptides identified through ex vivo phage display. In spite of these benefits, the access to fresh tissue samples is not easy for all research groups, tissue processing requirements yield a lower throughput compared to whole-cell biopanning, and the presence of multiple cell types in tissue samples causes heterogeneity versus more homogeneous cultured cells in whole-cell biopanning. These challenges can affect the reproducibility and consistency of the results of ex vivo phage display.

### 4.4. In Vivo Biopanning

Despite the promise of in vitro whole-cell and ex vivo biopanning, these selection schemes fail to fully capture the complex and varied environment of a living body, where factors like blood circulation, immune system activity, communications between organs, the diverse cellular populations, dynamic microenvironments, and in vivo physiological barriers all play important roles. In vivo phage display provides a more physiological platform for peptide selection by allowing phages to interact with the target within the full biological context of a living system [79]. In this selection modality, the phage library is injected into the tail vein of an animal and allowed to circulate for a short period, during which phage-displayed peptides can directly bind to specific targets on cells within tissues or organs. The animal then undergoes the perfusion of the left ventricle with saline to wash away unbound phages, followed by the euthanasia of the animal and the collection of the target tissues (e.g., xenograft tumors) or organs. These tissues are homogenized to recover bound phages. Phages retrieved from these homogenates are used to infect host bacteria for amplification. In the case of planning more than one round, the amplified phage pool is subsequently injected into another animal with the same genetic background. In vivo phage display is largely dependent on the more readily accessible vascular system of the target tissue or organ, and thus, the capillary vessels can pose a significant barrier for phage particles to traverse. Therefore, many peptides selected by using this approach tend to bind to endothelial cells of the vasculature of the target tissue or organ rather than the tissue or organ itself. Given the high degree of vascular heterogeneity among tissues, it is possible to isolate phages that bind to the differentially expressed cell surface proteins of endothelial cells in different tissues or organs [80]. By using in vivo phage display, peptides are selected in the complex physiological environment of an animal’s body with desired pharmacokinetic and pharmacodynamic characteristics. There are numerous studies in the literature reporting in vivo phage display in animal models, identifying a variety of vascular-targeting as well as tissue- or organ-targeting peptides [7,54,81,82,83,84,85,86]. However, in vivo phage display has rarely been reported in humans [81,87,88,89].

Each mode of the target presentation in phage display selection—recombinant protein, whole-cell, ex vivo, and in vivo systems—offers unique advantages and presents specific limitations. While recombinant proteins allow for high-throughput screening, they may lack physiological relevance. Whole-cell and ex vivo methods better preserve natural conformations but suffer from limitations in reproducibility or accessibility. In vivo biopanning, though powerful, is technically demanding and skewed toward vascular targets. Therefore, a combinatorial approach that integrates multiple presentation formats can significantly improve the identification of physiologically relevant, cell-selective peptides. Figure 3 provides a visual summary of the various modes of target presentation in the selection of phage display peptide libraries.

## 5. The Application of Phage Display for the Discovery of Cell-Selective Peptides

Targeting cell surface proteins is a powerful strategy for drug development [90,91]. Peptides are particularly promising tools for modulating these proteins. Here, we explore mechanisms by which peptides can interact with cell surface protein targets and then highlight the application of phage display technology for discovering cell-selective peptides. We aim to provide an overview of peptides identified over the past decade through the affinity selection of phage display libraries, highlighting the potential of this technology for developing therapeutic peptides for various diseases.

### 5.1. Peptides and Their Interactions with Cell Surface Proteins

Cell surface proteins play a crucial role in mediating biochemical and electrical communications between cells, enabling vital biological processes. Many transmembrane proteins and receptors have binding sites that are accessible to drugs, including peptides. The significance of these proteins and receptors in disease-associated cellular and molecular processes has created a huge interest in developing drugs targeting them [92,93,94]. Cell surface proteins do not act alone. They rely heavily on protein–protein interactions (PPIs) to transmit signals across the cell membrane. Advancements in omics technologies and structural biology have led to the discovery of many PPIs. PPIs play essential roles in regulating the mechanisms behind many cellular signaling pathways, and thus, their impaired regulation is a characteristic of many human diseases [95]. Cell surface-binding peptides hold potential as incredibly valuable tools in the development of therapeutics for a variety of diseases. Peptides might compete with the natural endogenous ligands to bind to the target cell surface protein and then interact with biologically relevant sites on the surface of the target molecule. Although this interaction involves a large contact surface, the majority of the binding energy is typically mediated by only a few key amino acid residues on both the ligand and the receptor. Upon the peptide binding, the cell surface protein or receptor undergoes conformational changes in the extracellular domain that enable or disable interactions with other molecules, such as proteins, triggering intracellular signal transduction pathways. Sometimes, the peptide interaction with the cell surface protein or receptor does not interfere with the binding of the native ligand, like peptides that bind to a monomeric receptor and prevent receptor oligomerization. For example, peptides derived from the transmembrane domains (TMDs) of G protein-coupled receptors (GPCRs) have been shown to disrupt receptor dimerization without affecting the binding of the natural ligand [96]. TMD peptides are short stretches of amino acids that can be discovered through screening approaches or designed rationally based on the structure or sequence of their target receptors, providing unique insights into receptor function and regulation [97]. Notably, a peptide can exhibit biological activity even without directly interacting with the receptor’s ligand binding site. For instance, it may act allosterically to modulate the receptor function, thereby affecting the binding of the endogenous ligand or the activity of the downstream signal transduction pathways [98].

Some membrane receptors are challenging targets for peptide drug discovery. In this context, GPCRs, which form one of the largest families of drug targets [99], have a complex structure that restricts their use as the target of biopanning. These receptors possess multiple transmembrane domains, and their extracellular ligand-binding region is composed of several distinct parts. Their structure is therefore highly dependent on preserving the integrity of the entire molecule, which is embedded within the cell membrane [98].

### 5.2. Cell Surface Protein-Binding Peptides Identified by Phage Display

Most of the approved peptide drugs are agonists, and peptide antagonists historically have faced a slower market entry. This is explained by the fact that agonists can achieve therapeutic effects by occupying only 5% to 20% of target receptor molecules, whereas antagonists must compete with the endogenous ligand and typically require a > 50% receptor occupancy for efficacy. In addition, peptide antagonists often function through allosteric receptor interactions, where their large surface area may offer minimal advantages over competing small-molecule drugs. Nevertheless, peptide antagonists demonstrate an obvious superiority over small molecules when the target requires an inhibitor with a large surface area and structural complexity (e.g., ion channels) to ensure subtype selectivity and minimize off-target effects [100]. Peptides targeting the erythropoietin receptor (EpoR) [101] and thrombopoietin receptor (TpoR) [102] are important examples of agonists of membrane receptors identified by phage display. In subsequent years, the TpoR-binding peptide underwent further modifications and was later developed into an FDA-approved drug. This drug, called romiplostim, is being used routinely in the clinic for the treatment of chronic immune thrombocytopenic purpura (ITP). Romiplostim is composed of two identical single-chain subunits, each containing a peptide with two TPOR-binding domains covalently linked to a human IgG1 Fc domain, forming a peptibody. The attachment to the Fc fragment increases the stability and half-life in the bloodstream [103,104].

Although only a small number of peptides identified through phage display have progressed to approved drugs, numerous candidates remain under investigation at various stages of research and development, holding significant potential as future therapeutics. Furthermore, the field itself continues to expand, with the scientific community actively identifying new peptides that exhibit promising biological activities. In particular, the increasing number of peptide candidates with known binding to cell surface proteins provides new opportunities for developing peptide-based therapeutics. Table 2 presents examples of cell surface-binding peptides identified by phage display over the past decade. These peptides have been reported to bind selectively to either cells or cell surface-associated proteins and receptors. Some of these phage-display-derived peptides might offer potential applications as therapeutic agents in the future.

## 6. Affinity Maturation of Peptides Derived from Phage Display by Building Secondary Libraries

Affinity maturation is a crucial step in peptide engineering that aims to improve the binding affinity and overall functionality of peptides identified through phage display. While the primary selection can yield candidate binders, these peptides often exhibit suboptimal affinities for their targets. To address this limitation, affinity maturation employs iterative cycles of mutation and selection to fine-tune peptide sequences for enhanced binding affinity. A central element of this process is the construction of secondary (and higher-order) libraries, which use initial peptide hits from a primary selection as the input for further diversification and selection [143]. In this section, we explore key strategies used in peptide affinity maturation, including the random approach and rational design. We then discuss how the number of peptide sequences selected as the input for constructing secondary and higher-order libraries influences affinity selection outcomes, comparing greedy and non-greedy strategies that can be employed to optimize the binding properties of peptides.

### 6.1. Definition and Scope of Affinity Maturation

A fundamental paradigm to improve the biological activity of peptides is affinity maturation. This strategy is mainly dependent on directed evolution and is classically used to enhance the binding affinity of a peptide. However, the underlying principles of affinity maturation—namely, iterative mutagenesis and selection—can also be employed to improve traits beyond binding affinity. These include pharmacokinetic and pharmacodynamic properties such as the peptide stability and solubility. In such cases, the maturation strategy focuses on optimizing these alternative traits, either within the phage display framework or during post-display peptide engineering. Due to the classical and predominant use of this strategy for the maturation of the binding affinity, here we focus on how this powerful approach can serve to refine the binding affinity of peptides toward a desired target. In a broad sense, affinity maturation is defined as a process in which iterative rounds of mutagenesis and selection are applied to enhance the binding affinity of a peptide that is specific for a given target. This concept is inspired by the natural immune response in antibody-encoding genes in B cells, where the somatic hypermutation and selection generate antibodies with a progressively increasing affinity for an antigen over time [144]. In the phage display context, the highest-affinity binders might be strongly underrepresented or entirely absent in the naïve library. This is of particular importance for longer peptides, as the coverage of their complete theoretical sequence spaces is beyond the capacities of the library construction methods. Additionally, biases that occur during the selection (e.g., inefficient recovery of tight binders) and the amplification of the selected pool (e.g., outcompeting high-affinity binders displayed on slow-propagating phages by low-affinity binders displayed on fast-propagating phages) can skew the selection toward the enrichment of weak binders. The net outcome is that initial hits identified in phage display selection may have suboptimal affinity. To address this challenge and enhance the probability of the discovery of the strongest binders, a secondary library can be constructed based on the sequence(s) identified in the primary selection [143]. At this stage, the peptides remain genetically encoded and displayed on the surface of phages, allowing the process of mutagenesis and selection to continue within the phage display system. It is only after high-affinity binders have been identified that peptide sequences are synthesized and tested independently for functional characterization or therapeutic development. In affinity maturation, randomization is performed on the most promising peptide hit(s) from a previous biopanning, followed by a secondary biopanning under a higher selection stringency. By doing so, the affinity of peptides that bind to the target in the micromolar range can be optimized to reach the nanomolar or even picomolar range [145].

### 6.2. Strategies for Peptide Affinity Maturation: Random Approach and Rational Design

**Figure 4:**
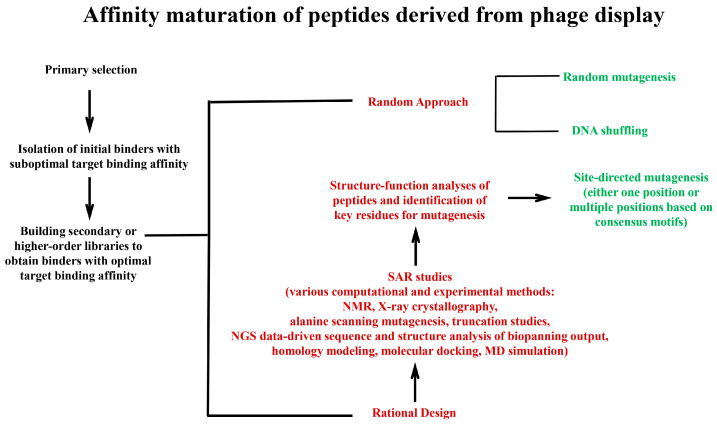
A schematic overview of the main strategies used for the affinity maturation of peptides derived from phage display selection. Peptides identified from the selection of phage display libraries exhibiting suboptimal affinities are used as the input for affinity maturation by building secondary or higher-order libraries. The diagram illustrates the two principal strategies for affinity maturation: the random approach and rational design. In the random approach, mutations are introduced into peptide sequences using stochastic methods such as random mutagenesis (e.g., error-prone PCR) and DNA shuffling, which generates secondary libraries that are then subjected to selection for enhanced binding affinity. In the rational design, structure–activity relationship (SAR) data, obtained from computational (e.g., molecular docking, molecular dynamics) and experimental methods (e.g., alanine scanning, truncation studies), provide insights into residues predicted to improve binding affinity upon modification, guiding the site-directed mutagenesis of these residues. The directed mutagenesis of multiple positions is enabled by analyzing the peptide pool of the biopanning output and identifying consensus sequence motifs in the peptide repertoire. In both strategies, the iterative cycles of mutagenesis and selection progressively improve binding affinity, with a potential extension to tertiary (and higher-order) libraries for further optimization.

Various strategies are employed for the affinity maturation of phage display-derived peptides, which can be classified into two broad categories of the random approach and rational design (Figure 4). The random approach involves the unbiased exploration of the vast sequence space of peptides through stochastic methods and introduces diversity into peptide sequences without prior knowledge of the structure–function relationship. The random approach includes techniques such as random mutagenesis [146] and DNA shuffling [147,148]. In random mutagenesis, random mutations are introduced into the peptide sequence (e.g., by error-prone PCR), whereas in DNA shuffling, sequences from different peptide variants are recombined to generate a library of chimeric peptides that might contain variants with enhanced binding characteristics.

In contrast, the rational design uses a combination of in silico and experimental methods to obtain peptides with an improved binding affinity in a targeted manner. In this context, structure–activity relationship (SAR) studies are highly important in systematically evaluating how structural changes affect the biological function as well as in identifying key residues involved in binding [149,150,151,152,153]. While SAR studies are often applied to peptides outside the phage display platform, they can inform rational design methodologies for constructing secondary libraries within the phage display system. In SAR studies, data obtained through nuclear magnetic resonance (NMR), X-ray crystallography, homology modeling, and computational tools play a pivotal role in the structure- and sequence-guided rational design of peptides to enhance the binding affinity without compromising their structural integrity or biological function. Computational methods like molecular docking and molecular dynamics (MD) simulations can be utilized to predict the best binding poses of a peptide to its target and identify residues with strong binding interactions between the peptide and the target, respectively [154]. Additionally, large sequence datasets generated by the NGS analysis of the biopanning output can also serve as a rich source to gain quantitative information about the abundance and enrichment of peptide variants during library selection, which can be effectively employed for data-driven peptide engineering. Experimental methods such as alanine scanning mutagenesis and truncation studies also play important roles in SAR studies. In alanine scanning mutagenesis, each amino acid of a peptide is replaced with alanine [155]. Residues that significantly reduce binding upon mutation are considered critical. Another type of SAR analysis is truncation study that involves removing a segment of a peptide to identify the essential amino acids or regions for binding [156]. The diverse information acquired from sequence- and structure-based analyses in SAR analyses provides valuable insights into key residues involved in binding. Based on these insights, residues predicted to enhance binding affinity when modified can be selectively targeted for mutagenesis. An important technique for the rational design of peptides for this purpose is site-directed mutagenesis [157]. The candidate residues are targeted by this method, either to introduce a specific amino acid substitution or to explore multiple alternatives, sometimes even all 19 other amino acids. In some cases, sequence and structural analyses suggest that multiple positions should be selected for mutagenesis to achieve optimal results. In this regard, the consensus motifs identified by the analysis of the results of biopanning data can be used to design secondary focused libraries for the affinity maturation of phage display-derived peptides [158,159,160]. A consensus motif is a recurring sequence pattern observed among a group of peptides that have been identified in biopanning against a specific target. The highly conserved residues of a consensus motif are used as a scaffold and thus kept intact (fixed positions), whereas semi-conserved or variable positions are mutated for further optimization. The motifs can help in identifying binding hotspots, regions on the peptide that interact most strongly with the target. Secondary focused libraries constructed based on consensus motifs preserve the core structure of the parental binder while permitting the fine-tuning of specific residues to improve binding affinity. In some cases, the combinatorial optimization of motifs can occur by merging multiple consensus motifs identified in the same or different selections. Figure 4 is a schematic diagram of the strategies used for the affinity maturation of phage display-derived peptides.

The process of the evolution of the binding affinity maturation of peptides can be further extended beyond primary (first-generation) and secondary (second-generation) libraries by constructing tertiary (third-generation) libraries. Tertiary libraries are generated based on the input from secondary libraries. This iterative process holds potential for the gradual improvement of the binding affinity of peptides. Such a stepwise evolutionary selection strategy has been used to construct phage display secondary and tertiary sub-libraries to identify peptides that bind to and inhibit hemagglutinin (HA) (the membrane protein of type A influenza viruses). The selection from secondary and tertiary sub-libraries led to an enhanced binding affinity of peptides and narrowed down the minimum inhibitory sequence to a 5-residue motif critical for inhibition. These HA inhibitors are considered promising candidates for the development of antiviral drugs. It is of interest to note that the selection and optimization process, including the construction of secondary and tertiary sub-libraries, was carried out within the phage display system. However, the most promising candidates were later synthesized and tested as free peptides to confirm their inhibitory activity and therapeutic relevance [161].

### 6.3. Greedy vs. Non-Greedy Strategies for Optimization of Peptide Binding Affinity

**Figure 5:**
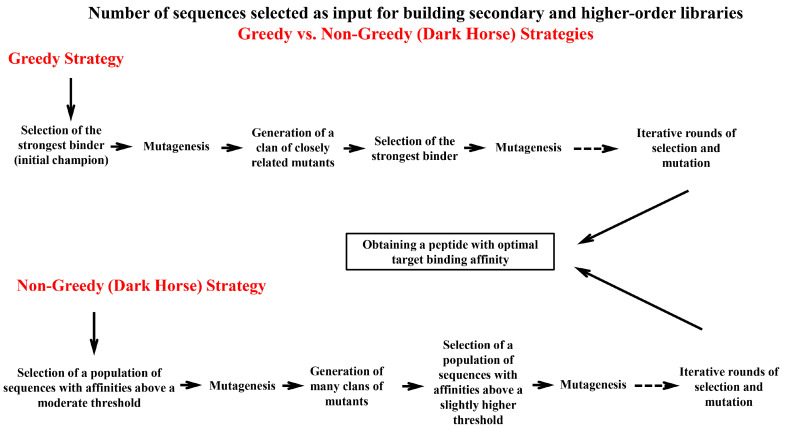
A comparison of greedy and non-greedy strategies for the selection of input sequence(s) when building secondary and higher-order libraries. The diagram compares the greedy (**top**) and non-greedy (**bottom**) strategies for the optimization of binding affinity. In the greedy strategy, the strongest binder (initial champion) from the primary selection is selected and subjected to mutagenesis. This process is repeated for further cycles of mutagenesis and selection, leading to a localized exploration of the sequence space (in the neighborhood of only the first-best sequence). This localized search risks missing superior binders in distant, unexplored regions of the sequence space (e.g., the neighborhoods of the second-best sequence, third-best sequence, and so on). In contrast, the non-greedy strategy retains a broader selection window by including a subpopulation of binders above a moderate affinity threshold during each cycle of selection and mutagenesis. This strategy is capable of capturing dark horse sequences that may be inferior to the strongest binder in terms of target binding affinity but hold the potential to surpass the affinity of the strongest binder through further rounds of mutagenesis and selection. By iteratively increasing the selection stringency during cycles of in vitro evolution and including a population of sequences with affinities slightly higher than the previous round, the non-greedy strategy enables the simultaneous exploration of multiple sequence neighborhoods. Broadening the exploration of the sequence landscape enhances the probability of discovering peptides with optimal target binding affinity.

Regarding the number of peptide sequences selected as the input for building secondary and higher-order libraries, two main strategies might be employed (Figure 5). The first one, which is known as the greedy strategy, involves selecting the best clone (the strongest binder), called the initial champion, and then subjecting this clone to either random or rational mutagenesis. This generates a clan of closely related variants. Again, the best binder from this clan is selected and mutagenized. This alternating process of selection and mutation is repeated until a peptide with optimal binding for the target is obtained. In this optimization program, each iteration selects “greedily” for the very best binder available within the population [162]. A significant limitation of this optimization scheme is that only close relatives of the initial champion are explored. Therefore, a minuscule region within the vast space of all possible sequences is searched to find the most fit sequence(s). However, it is entirely possible this localized search in the neighborhood of only the first-best sequence overlooks superior sequences that reside in distant, unexplored regions of the sequence space, including the neighborhoods of the second-best sequence, third-best sequence, fourth-best sequence, and so on. As a consequence, a more thoughtful alternative to the greedy plan is to relax the stringency of earlier selection round(s) to search in a broader part of the possible sequence space for finding more fit sequences. Based on this, rather than selecting the very best clone in the current population, a broader subpopulation of clones is selected that exhibits affinities above a certain moderate threshold. This baseline threshold is sufficiently permissive to retain “dark horses”, variants in the output of the primary selection that are (somewhat) inferior to the initial champion but possess the potential to be mutated and reach fitness levels even higher than the initial champion. In this non-greedy binding optimization strategy, a cocktail of sequences with diverse fitness levels (above the defined baseline level), containing thousands or millions of different variants, is selected and subjected to mutagenesis en masse, generating many clans of mutants. In the next round, a mixture of sequences with broad fitness levels (above a slightly higher threshold than the previous round) is selected from different clans of mutants. This process is repeated while increasing the selection stringency with each successive round. At the end of the final round, a stringent selection is employed to select the very best sequence in the existing population. By applying this scheme, dark horse sequences that reside in a different neighborhood than the initial champion can be discovered [162,163]. When sequences are similar and lie in the same neighborhood in the sequence space, they often converge on the same local optimum through mutational trajectories. However, the non-greedy strategy provides the opportunity to explore dark horse sequences from other neighborhoods of the sequence space, increasing the probability of finding the very best sequence that could exist in the possible sequence space. It is important to note that even by using the non-greedy strategy, it is not possible to explore the entire sequence space, and there is always the chance that a significantly better binder exists somewhere in the vast expanse of the sequence space. However, considering the impracticality of conducting a truly global search, the close neighborhoods of the best binder in the sequence space remain the most promising areas to become the focus of ligand optimization efforts [162]. Figure 5 illustrates a schematic comparison of the greedy and non-greedy strategies for optimizing the binding affinity of phage display-derived peptides.

## 7. Concluding Remarks and Future Perspectives

Over many years, phage display has proven to be a reliable and powerful method for finding peptides that bind to a wide variety of clinically relevant targets [164,165]. One of the most transformative contributions of phage display has been its role in revolutionizing the discovery of cell surface-binding peptides. By enabling the selection of peptides that recognize specific molecular signatures on the surface of cells—either through purified targets or complex biological systems such as whole cells, tissues, and living organisms—phage display has fundamentally reshaped the way targeting ligands are developed. This has had wide-reaching implications for drug delivery, molecular imaging, and the design of precision therapeutics. In addition, the expanding use of phage display in complex biological environments, such as ex vivo and in vivo settings, represents the promise of selecting peptides with a high physiological relevance. These applications have evolved phage display from a pioneering display technology into a cornerstone of modern pharmaceutical research.

The success of phage display depends not only on the construction of diverse and well-designed peptide libraries but also on the strategic presentation of targets and the optimization of affinity selection protocols. Importantly, the transition from peptide discovery to therapeutic application necessitates improvements in binding affinity and pharmacological properties. The development of secondary libraries, constructed by either random approach or rational design through SAR studies, serves as a crucial step in maturing peptides toward clinical relevance. Several technical innovations have made phage display a more powerful strategy for peptide discovery. In this context, advancements in phage display technology have enabled the construction of sophisticated peptide libraries with topologically constrained formats. Among these, bicyclic and multicyclic peptide libraries represent a major innovation, offering an enhanced conformational rigidity, optimized resistance to proteolytic degradation, and improved receptor-binding affinity compared to their linear or even monocyclic counterparts [166,167,168]. The structural constraints imposed by multiple cycles allow these peptides to mimic protein-like interfaces more effectively, facilitating high-affinity and selective interactions with challenging biological targets, such as protein–protein interaction surfaces or cryptic binding pockets in cell surface proteins. Moreover, expanding the chemical diversity of displayed peptides through genetic code expansion by introducing noncanonical amino acids with novel reactivities and chemistries has opened new avenues for improving the stability and specificity of peptides identified by phage display [169,170]. The incorporation of NGS into phage display in recent years has further enhanced the precision and throughput of this technology in peptide discovery [56,57], allowing for a deeper exploration of peptide repertoires and more informed decision-making during lead optimization.

Looking ahead, several areas offer exciting potential for further innovation in phage display. The integration of AI and ML into phage display workflows is poised to radically change the discovery of target-binding peptides [52,171]. The rapid expansion of sequencing data generated by NGS-based phage display has created a fertile ground for the application of AI and ML tools. By analyzing large-scale sequencing data from NGS, AI and ML algorithms can uncover hidden sequence–function relationships, predict the peptide-target binding affinity, cluster functionally relevant motifs, and prioritize candidates for further testing—far beyond what traditional methods can achieve. Additionally, these tools can guide the design of secondary libraries by predicting beneficial mutations or identifying underrepresented yet functionally promising “dark horse” candidates. As these computational approaches continue to mature and integrate with experimental workflows, they will increasingly complement wet-lab methods, enabling the faster, smarter, and more efficient identification of cell-selective peptide ligands with diagnostic and therapeutic potential. AI-assisted computational approaches are transforming how we interpret, navigate, and exploit the vast sequence space explored during biopanning.

Despite the promise of phage display in identifying cell-selective peptides, using this technology is not without restrictions. One of the most significant and technically challenging limitations of phage display, particularly when applied to complex biological targets such as the cell surface, is the undesirable enrichment of nonspecific binders, which can significantly compromise the efficiency and specificity of the selection process [172]. These nonspecific binders can broadly be categorized into two types: selection-related nonspecific binders, which interact with off-target components present in the selection system (e.g., plastic surfaces, blocking buffer), and propagation-related nonspecific binders, which are enriched not because of target selectivity but due to intrinsic propagation advantages that favor their amplification during biopanning rounds [173]. This phenomenon distorts the apparent outcome of selection, allowing peptides that do not specifically bind to the intended target to dominate the biopanning output. These issues are particularly challenging in cell-based biopanning, where the heterogeneity of the cell surface presents a significant technical barrier. Mammalian cell surfaces are highly complex and dynamic mosaics composed of a wide variety of biomolecules. This heterogeneity makes it difficult to identify peptides that bind selectively and reproducibly to a unique molecular marker, especially given the short length of peptides, which limits their interaction surface area and structural complexity. Further compounding the issue is that the molecular identity of the peptide’s target is often unknown or undefined in cell-based selections [174], which can severely hinder the downstream validation, mechanistic elucidation, and functional interpretation. Peptides isolated through cell-based biopanning may bind to a wide range of possible targets. Additionally, cell culture artifacts pose a substantial challenge. Cells grown in vitro often exhibit gene expression patterns, membrane compositions, and glycosylation profiles that diverge significantly from their native tissue context, leading to the enrichment of peptide binders that lack physiological relevance. Addressing these challenges requires the rigorous incorporation of counter-selection strategies and the utilization of robust bioinformatics pipelines capable of filtering out selection-related nonspecific peptides and propagation-biased sequences during data analysis. Nevertheless, the isolation of nonspecific binders remains a critical limitation that must be carefully managed to fully realize the promise of phage display in discovering functionally relevant, high-affinity cell-selective peptides.

Using the full potential of phage display for the discovery of cell-selective peptides will depend on addressing its major limitations. However, this molecular display framework remains not only a foundational technology in peptide discovery but also a continually evolving platform that adapts to the needs of modern biomedical research. Its synergy with increasingly sophisticated selection methodologies, chemical innovation, and computational tools will further enhance our capacity to discover and optimize high-affinity cell surface-binding peptides, bringing us closer to a future of more precise, effective, and personalized therapies.

## Figures and Tables

**Table 1:** The relationship between the number of fully randomized positions and the percentage of the whole sequence space that can be represented by a phage display random peptide library (known as the coverage percentage). The total number of possible sequences (theoretical sequence space) is calculated as 20^N^, where N is the number of fully randomized positions in the peptide library. The coverage percentage is determined by dividing 10^9^, which is the maximum achievable diversity in phage display peptide libraries, by the theoretical sequence space (the 2nd column). In practice, the maximum diversity of 10^9^ is difficult to reach, and thus, the coverage percentage is very likely to be lower than what is shown in the table. The full randomization is unbiased, meaning that each position of the library is allowed to vary completely, using all 20 possible amino acids. However, in the case of a partial randomization in which the variation in each position of the library is biased toward a subset of, not all, amino acids, the possible sequence space becomes smaller, and the coverage percentage represented by the library is significantly increased. Partial randomization is applied to build secondary libraries (described in Section 6).

Number of Fully Randomized Positions	Number of Possible Sequences	Coverage Percentage
6	6.4 × 10^7^	100
7	1.28 × 10^9^	78.125
8	2.56 × 10^10^	3.90625
9	5.12 × 10^11^	0.1953125
10	1.024 × 10^13^	0.009765625
11	2.048 × 10^14^	0.00048828125
12	4.096 × 10^15^	0.0000244140625
13	8.192 × 10^16^	0.000001220703125
14	1.6384 × 10^18^	0.00000006103515625
15	3.2768 × 10^19^	0.0000000030517578125
16	6.5536 × 10^20^	0.000000000152587890625
17	1.31072 × 10^22^	0.00000000000762939453125
18	2.62144 × 10^23^	0.0000000000003814697265625
19	5.24288 × 10^24^	0.000000000000019073486328125
20	1.048576 × 10^26^	0.00000000000000095367431640625

**Table 2:** A list of peptide sequences binding to cells or cell surface proteins identified in the recent decade from phage display libraries. The table indicates the sequence of isolated peptide(s), the target of the library selection, the involved disease, information about peptide properties and their biological function, and the publication reporting the peptide(s). In these reports, the target is a cell surface biomolecule that is presented to the library in different modes: a purified recombinant protein as well as a protein on the surface of either cultured cell lines (in vitro biopanning) or xenograft animal models of the relevant cell (in vivo biopanning). The presentation mode of the targets is noted in the table for each study. All targets (recombinant proteins and cells) are of human origin, unless otherwise noted. The references are arranged in the table in reverse chronological order (from newest to oldest).

Peptide Sequence(s)	Target	Indication	Notes	Reference
LSPLIFVTTPDT	B16 C-C chemokine receptor type 7 (CCR7) cells	Various cancers	D-amino acid was introduced into the peptide to increase its resistance to proteolysis. The modified peptide blocked the CCR7 pathway, inhibiting tumor growth and tumor lymph node metastasis in vivo. The Kd of the modified peptide with mCCR7-EGFP fusion protein was 403 nM, but without interaction with the control EGFP protein, it was >1000 nM.	[105]
SQHWTQASTARS SYDQRNFSQIRY	HeLa cells transfected with latent membrane protein 1 (LMP1), synthetic peptides representing the LMP1 extracellular domain, and Balb/C male mice intravenously injected with the phage output from in vitro biopanning	LMP1-overexpressing malignancies, such as nasopharyngeal carcinoma (NPC)	The interaction energy score was determined by molecular docking. SQHW exhibited an interaction energy of −8.6 kcal/mol, and SYDQ showed an interaction energy of −8.9 kcal/mol.	[106]
FKQDAWEAVDIR DSSPRMWPNRIT	HeLa cervical cancer and MDA-MB-231 breast cancer cells	Breast and cervical cancers	Peptides were selected by combining phage display with in silico molecular docking analysis. Peptides were suggested to bind to common receptors on both cell lines. The interaction energy score calculated by docking was −551.26 for FKQD and −916.32 for DSSP.	[107]
HHGANSLGLVQS YALGRPSLQGPN	Mouse Leydig cells (TM3)	Testicular disorders (such as male infertility)	The output of in vitro biopanning was used as input for in vivo biopanning in a mouse model. Phage display was combined with NGS. Peptides indicated accumulation in the mouse testis in vivo.	[108]
YLASRVH	Leucine-rich repeat-containing G-protein coupled receptor 5 (LGR5) recombinant protein	Gastric cancer	The fluorescently labeled cyclic peptide exhibited high accumulation in a gastric cancer xenograft mouse model.	[109]
HPDMFTRTHSHN	Epithelial cell adhesion molecule (EpCAM) recombinant protein	Hepatocellular carcinoma (HCC)	The peptide labeled with an NIR fluorophore exhibited high uptake in orthotopic human HCC patient-derived xenograft (PDX) tumor as well as local and distant metastases. The apparent Kd of the labeled peptide to Hep3B HCC cells was 67 nM.	[110]
NTGSPYE	Human gastric cancer xenograft tumor (vasculature) in nude mice	Gastric cancer	In vitro negative screening was combined with in vivo positive screening. The cyclic peptide exhibited selective accumulation in the vasculature of gastric cancer in vivo.	[111]
VLGREEWSTSYW	Epidermal growth factor tyrosine kinase receptor mutation variant III (EGFRvIII) recombinant protein	Various cancers	Phage display was combined with NGS. The estimated Kd of the peptide for the target was 361.5 ± 1172.0 µM. The docking score of the peptide was −186.5 kcal/mol.	[53]
HAMRAQP	SW480 colon adenocarcinoma cells	Colon cancer	A rigorous negative selection was applied before positive selection by exposing the naïve library to an empty well, a serum-treated well, and multiple control cells.	[64]
LNTPLKS	Mouse pancreatic islet β cell line MIN6	Diabetes	The FITC-labeled peptide exhibited specific accumulation in the tumor in the insulinoma animal model.	[112]
LSMPWSPTTYAS	Insulin-like growth factor 2 mRNA-binding protein 2 (IGF2BP2) recombinant protein	Esophageal squamous cell carcinoma (ESCC)	The NIRF-conjugated peptide exhibited high tumor accumulation in KYSE-30-bearing esophageal cancer xenograft animal models.	[113]
HVPGSYI VNAMQSY	Three types of EGFR- expressing cells derived from non-small cell lung cancer (NSCLC) (H1299 and H1297) and glioblastoma (DKMG)	Various cancers	Phage display was combined with NGS. The cyclic peptide conjugated to camptothecin (CPT) indicated toxicity in EGFR-overexpressing cells. In silico docking showed binding of peptides at the active site of EGFR. The binding energies of peptides to EGFR were −13.0 kcal/mol for HVPG and −11.9 kcal/mol for VNAM.	[114]
DPFYSMLQRLAH	MCF-7 breast cancer cells	Breast cancer	Bioinformatics analysis suggested that the peptide targets 5-lipoxygenase-activating protein (FLAP), involved in breast cancer progression through arachidonate metabolism.	[115]
GLEASRHPHGSW GDGNSVLKPGNW AMSDHHWTQRDK (Pep-39 as the most potent PD-L1 inhibitor)	Programmed death-ligand 1 (PD-L) recombinant protein	Various cancers	In-solution panning strategy using magnetic beads was used. All three peptides had interactions with PD-L1 in the vicinity of the PD-1 binding site. One of the peptides exhibited inhibitory potential against PD-1/PD-L1 interaction, reducing the survival of MDA-MB-231, CT 26, and DU-145 cells. The binding energy of this peptide, determined through docking analysis, was −35.5 kcal/mol for van der Waals and −130.8 kcal/mol for electrostatic interactions.	[116]
F3 peptide (peptide sequence not mentioned in the paper)	Glypican-3 (GPC-3) recombinant protein	Hepatocellular carcinoma (HCC)	The cyclic peptide indicated high accumulation in HepG-2 tumors in xenograft mouse models. The peptide labeled with ^68^Ga, the peptide tracer, enabled the specific detection of tumors in HCC tumor models with PET imaging.	[117]
PPRRGLIKLKTS	Human immortalized epithelial-like ectopic endometriotic cells (12Z) and human immortalized eutopic endometrial stromal cells (HESC)	Endometriosis	The peptide promoted the penetration and cytotoxicity of silver nanoparticles (AgNPs) in endometriotic spheroids.	[118]
ANLNLWTDYIRW	Colon cancer cells	Colon cancer	The peptide was conjugated to hematoporphyrin, a photosensitizer, which showed a significantly enhanced cellular uptake and high photodynamic effect to kill tumor cells. A nanoparticle modified with the peptide delivered SN-38 (an anti-cancer drug) into tumor cells, and its targeting ability was observed in vivo after intravenous injection into xenograft animal models. Structural modeling and MD simulation showed that the peptide is expected to form an amphipathic α-helix conformation, leading to its strong cell attachment.	[119]
MC1 peptide (peptide sequence not mentioned in the paper)	Pancreatic cancer (Mia Paca-2) cells	Pancreatic cancer	Phage display was combined with NGS. Two peptides had the EC_50_ values of 16.11 M and 97.01 M, suggesting them to be appropriate for detection and imaging purposes.	[120]
PSPHRQRQHILR QTIRIIIRRSRT SLHMRHKRKPRR SSRSMQRTLIIS	Patient-derived brain tumor initiating cells (BTICs)	Glioblastoma	Cargo-conjugated peptides delivered contrast-enhancing agents to highly infiltrative tumor populations in intracranial xenograft models without the obvious need for blood–brain barrier (BBB) disruption. The peptides could cross the BBB and home to their respective cellular targets in vivo. Simultaneous use of five independent targeting peptides provided greater coverage of this complex tumor, and selected peptides had the capacity to deliver a therapeutic cargo (oncolytic virus VSVΔM51) to the tumor cells in vivo. Gadolinium-peptides enhanced MRI of compact and diffuse GBMs in vivo.	[121]
ELTVMGYYPGMS	HEK-293 cells overexpressing CD44v6	Gastric cancer	The Kd of the peptide to the target protein was 611.2 nM. The FITC-labeled peptide accumulated in tumors in subcutaneous GC xenograft models.	[122]
SLSHSPQ	Human cerebral microvascular endothelial cell line (hCMEC/D3 cells)	A model for permeability/delivery across the human blood–brain barrier (BBB)	Phage display was combined with a transcellular permeability assay. The cyclic peptide facilitated BBB permeation of M13 phage using a transcellular permeability assay with hCMEC/D3 cell monolayers (a human BBB model). Phage-peptide internalization into monolayer cells was suggested to be mediated via receptor-mediated macropinocytosis. Peptide–phage distribution into the brain parenchyma was observed in mice after intravenous administration. Furthermore, liposomes functionalized with the peptide permeated across the BBB in mice in vivo.	[123]
WRARVPL	Fibroblast growth factor receptor 2 (FGFR2)	Various cancers	The peptide might possess the potential to be an inhibitor for FGFR2. The Kd of the peptide for the target protein was ≈1.4 µM.	[124]
SFKIPYHYDSGQ	Endothelial progenitor cells (EPCs)	Increasing EPC proliferation and reducing thrombogenicity (thrombus formation)	Phage binding index (PBI) was used to evaluate the quality as well as to measure the target affinity of selected peptides. The peptide was able to reduce platelet activation and decrease thrombus formation.	[125]
AWRTHTP	A549 non-small cell lung carcinoma cells	Lung cancer	A rigorous negative selection was used by exposing the naïve unselected library to an empty well, a serum-treated well, and multiple control cells before positive selection.	[126]
DWSSWVYRDPQT	COLO320HSR colon cancer cells	Colon cancer	Bioinformatics analyses suggested that the peptide targets human glypican-3, which is involved in the development of multiple cancer types.	[127]
QVNGLGERSQQM	HeLa cervical cancer cells with high CD55 expression	Cervical cancer	The peptide could bind to CD55 on the surface of HeLa and SiHa cells. It could also effectively inhibit the proliferation and induce apoptosis in several cervical cancer cells. The IC50 values of the peptide on SiHa and HeLa cells were 208.4 ± 13.5 µg/mL and 230.3 ± 20.1 µg/mL, respectively.	[128]
GDALFSVPLEVY FTPGGNTYAGQP SIDDQRDVGEWG KQNLAEG	Cervical cancer xenograft model in mouse	Cervical cancer	Peptides exhibited tumor targeting in cervical cancer xenograft mouse models.	[129]
MHTAPGWGYRLS	Folate receptor alpha (FRα) recombinant protein	Various epithelial cancers (such as ovarian cancer)	The peptide exhibited tumor targeting in vivo by both phage homing experiment (phage-displayed peptide) and fluorescence imaging (synthetic peptide labeled with FITC) in ovarian cancer xenograft mouse models. The Kd of the peptide for the target protein was 0.3 µM. It could be internalized into SKOV3 cells. Computational docking analysis indicated that amino acids at the C-terminus of the peptide form more stable hydrogen bonds with the target protein, and the peptide could bind to the target at the entrance of the folate-binding pocket, but does not stick into the pocket.	[130]
DNPGNET	Caco-2 colon carcinoma cells	Colon cancer	The Kd of the peptide for Caco-2 cells was around 10 µM. The peptide could facilitate transcellular permeation of phages across a Caco-2 cell monolayer in vitro due to reduced cell viability arising from cytotoxicity and/or opening of the tight junctions between cells, as well as the permeability of phages across mouse intestinal epithelium. The transcellular transport of peptide–phage was suggested to be mediated by micropinocytosis, with the involvement of the αvβ3 receptor.	[131]
ASSHN	Human endothelial progenitor cells (EPCs) and angiogenesis mouse model prepared by the dorsal air sac (DAS) method	Various cancers (tumor angiogenic vessels)	A combined in vitro and in vivo biopanning was conducted. Peptide-modified liposomes could accumulate in tumor tissue in vivo, likely via binding to tumor vessels and the EPR effect. Also, peptide-modified liposomes carrying doxorubicin significantly reduced tumor growth in Colon26 NL-17-bearing mice.	[132]
YIAPPHTSEDSN	HuCCT-1 human cholangiocarcinoma cells	Bile duct cancer	On-chip phage display screening was performed on the integrated microfluidic chip using magnetic beads. The Kd of the fluorescently labeled peptide toward HuCCT-1 was 3 µM.	[133]
QQLPSSSTSTYP	SiHa human cervical cancer cells	Cervical cancer	Cell immunofluorescence assay indicated binding of the peptide to the membrane of SiHa cells.	[134]
GAMHLPWHMGTL NPWEEQGYRYSM NNPWREMMYIEI	H460 large cell carcinoma (LCC) cells	Lung cancer	All peptides specifically bound to lung cancer cells and exhibited tumor-homing ability in vivo. Kd values were as follows: ~5.72 µM for GAMH peptide, ~12.5 µM for NPWE, and ~5.52 μM for NNPW peptide. Liposomal doxorubicin conjugated to the peptides exhibited great therapeutic efficacy in orthotopic lung cancer animal models. In vivo optimal imaging of phage homing and MRI of peptide–super-paramagnetic iron oxide nanoparticles (SPIONs) indicated that GAMH peptide is the most favorable probe for multimodal molecular imaging, while NPWE and NNPW peptides significantly improved intracellular drug delivery in vivo due to their endocytosis, resulting in longer overall survival in treated mice.	[135]
PRWAVSP DTFNSFGRVRIE	MDA-MB-231 human breast cancer (claudin-low breast cancer)	Claudin-low breast carcinomas	Phage display selection was performed through direct coating and BRASIL methods. Bioinformatics analyses indicated that the PRWA peptide could bind to metalloproteinase inhibitor 1 (TIMP-1), and the DTFN peptide could bind to plasminogen activator inhibitor 1 (PAI1) precursor, both related to breast cancer. Docking of peptides against the predicted binding partners indicated the lowest energy weighted score of −1127 for TIMP-1 and −1046.5 for PAI1.	[136]
GSTSFSK	HepG2 hypoxic hepatoma cells and hypoxic HepG2-made hepatocarcinoma model in mouse	Hepatocellular carcinoma	A combined in vitro and in vivo panning was used. The peptide indicated a clear selectivity toward the tumor tissue in HCC tumor-bearing mice.	[137]
NPMIRRQ	HO-8910 human ovarian cancer cells	Ovarian cancer	Peptide binding to HO-8910 cells was confirmed by immunocytochemical and immunohistochemical staining.	[138]
GYSASRSTIPGK	Breast cancer stem cells (isolated from the MDA-MB-231 cell line)	Breast cancer	Target cells were isolated using the serum-free suspension culture technique, resulting in minimal damage to the isolated stem cells. Dual-subtract biopanning was applied by using Hs 578Bst and MDA-MB-231 for library subtraction.	[139]
THENWPA	CD44v3-v10 recombinant protein	Gastric cancer (CD44v-positive stomach tumors)	Subtractive biopanning was performed by incubating the phage library with bovine serum albumin (BSA) and CD44.	[140]
GTIQPYPFSWGY	Prostate-specific membrane antigen (PSMA) recombinant protein, PSMA-positive LNCaP cells, and LNCaP xenografts in nude mice	Prostate cancer	The apparent Kd values of the peptide for PSMA-positive LNCaP and C4-2 cells were 8.22 µM and 8.91 µM, respectively. The peptide could specifically deliver the proapoptotic peptide _D_(KLAKLAK)_2_ to LNCaP cells to induce cell death. In biodistribution studies, the peptide indicated the highest uptake in human prostate xenograft tumors in mouse models.	[141]
TTMPIDS TTPTKSA	HEK 293 cells expressing the α1β glycine receptor	Inflammatory pain and alcoholism	A negative selection was performed on HEK 293 cells expressing alternative glycine receptor subtypes before positive selection on HEK 293 cells expressing the α1β subtype. The peptides might present potential for potentiating the receptor function, acting as allosteric enhancers.	[142]

## Abbreviations

AgNPs	Silver nanoparticles
AI	Artificial intelligence
BBB	Blood–brain barrier
BTIC	Brain tumor initiating cell
BSA	Bovine serum albumin
CCR7	C-C chemokine receptor type 7
CPT	Camptothecin
DAS	Dorsal air sac
ECM	Extracellular matrix
EGFRvIII	Epidermal growth factor tyrosine kinase receptor variant III
EPC	Endothelial progenitor cell
EpCAM	Epithelial cell adhesion molecule
EpoR	Erythropoietin receptor
ESCC	Esophageal squamous cell carcinoma
FGFR2	Fibroblast growth factor receptor 2
FLAP	5-lipoxygenase-activating protein
FRα	Folate receptor α
GPCR	G-protein coupled receptor
GPC-3	Glypican-3
GST	Glutathione S-transferase
HA	Hemagglutinin
HAE	Hereditary angioedema
hCMEC	Human cerebral microvascular endothelial cell
HCC	Hepatocellular carcinoma
HESC	Human immortalized epithelial-like eutopic endometrial stromal cell
\	
HTS	High-throughput sequencing
HuCCT-1	Human cholangiocarcinoma cell line T1
IGF2BP2	Insulin-like growth factor 2 mRNA-binding protein 2
ITP	Immune thrombocytopenic purpura
LCC	Large carcinoma cell
LGR5	Leucine-rich repeat-containing G-protein coupled receptor 5
LMP1	Latent membrane protein 1
MBP	Maltose-binding protein
MD simulation	Molecular dynamics simulation
ML	Machine learning
MMP	Matrix metalloproteinase
NGS	Next-generation sequencing
NMR	Nuclear magnetic resonance
NPC	Nasopharyngeal carcinoma
NSCLC	Non-small cell lung cancer
OPA	Ortho-phthalaldehyde
PAI1	Plasminogen activator inhibitor 1
PBI	Phage binding index
PD-L1	Programmed death-ligand 1
PDX	Patient-derived xenograft
pIII, pVI, pVII, pVIII, pIX	Coat proteins (3, 6, 7, 8, 9) of the filamentous M13 phage
PNH	Paroxysmal nocturnal hemoglobinuria
PPI	Protein–protein interaction
PSMA	Prostate-specific membrane antigen
SAR	Structure–activity relationship
SPIONs	Super-paramagnetic iron oxide nanoparticles
ssDNA	Single-stranded DNA
TBMB	1,3,5-tris(bromomethyl)benzene
TIMP-1	Tissue inhibitor of metalloproteinases 1 (metalloproteinase inhibitor 1)
TMDs	Transmembrane domains
TpoR	Thrombopoietin receptor
TUPs	Target-unrelated peptides
